# Efficient bioremediation of PAHs-contaminated soils by a methylotrophic enrichment culture

**DOI:** 10.1007/s10532-022-09996-9

**Published:** 2022-08-17

**Authors:** Kartik Dhar, Logeshwaran Panneerselvan, Kadiyala Venkateswarlu, Mallavarapu Megharaj

**Affiliations:** 1 Global Centre for Environmental Remediation (GCER), College of Engineering, Science and Environment, The University of Newcastle, ATC Building, University Drive, Callaghan, NSW 2308 Australia; 2 Cooperative Research Centre for Contamination Assessment and Remediation of the Environment (CRC CARE), The University of Newcastle, ATC Building, Callaghan, NSW 2308 Australia; 3 Formerly Department of Microbiology, Sri Krishnadevaraya University, Anantapuramu, 515003 India

**Keywords:** Polycyclic aromatic hydrocarbons (PAHs), Bioremediation, Methylotrophic bacteria, Enrichment culture, *Rhizobiaceae*, *S*oil slurry

## Abstract

Bioaugmentation effectively enhances microbial bioremediation of hazardous polycyclic aromatic hydrocarbons (PAHs) from contaminated environments. While screening for pyrene-degrading bacteria from a former manufactured gas plant soil (MGPS), the mixed enrichment culture was found to be more efficient in PAHs biodegradation than the culturable pure strains. Interestingly, analysis of 16S rRNA sequences revealed that the culture was dominated by a previously uncultured member of the family *Rhizobiaceae*. The culture utilized C1 and other methylotrophic substrates, including dimethylformamide (DMF), which was used as a solvent for supplementing the culture medium with PAHs. In the liquid medium, the culture rapidly degraded phenanthrene, pyrene, and the carcinogenic benzo(a)pyrene (BaP), when provided as the sole carbon source or with DMF as a co-substrate. The efficiency of the culture in the bioremediation of PAHs from the MGPS and a laboratory waste soil (LWS) was evaluated in bench-scale slurry systems. After 28 days, 80% of Σ16 PAHs were efficiently removed from the inoculated MGPS. Notably, the bioaugmentation achieved 90% removal of four-ringed and 60% of highly recalcitrant five- and six-ringed PAHs from the MGPS. Likewise, almost all phenanthrene, pyrene, and 65% BaP were removed from the bioaugmented LWS. This study highlights the application of the methylotrophic enrichment culture dominated by an uncultured bacterium for the efficient bioremediation of PAHs.

**Supplementary Information:**

The online version contains supplementary material available at 10.1007/s10532-022-09996-9.

## Introduction

Manufactured gas plants (MGP) were operational in Australia from the late 1800s to the 1960s. Although decommissioned a long ago, many of the former MGP sites are contaminated with chemicals generated during the town gas production process. As a result, many former MGP sites will need remediation before repurposing the lands to meet increasing land demand. Polycyclic aromatic hydrocarbons (PAHs) are significant contaminants of concern in MGP sites (Kuppusamy et al. 2017; Larsson et al. 2018; Cao et al. 2020). Due to their toxic and carcinogenic potentials, PAHs pose serious risks to the environment and human health (IARC 2010). Remediation of PAHs-contaminated MGP soils is challenging mainly due to the long contamination history and weathering process, and the remarkable persistence of the chemicals. Several treatment technologies such as incineration, soil washing and chemical oxidation are available for the remediation of PAHs-contaminated soils (Kuppusamy et al. 2017; Sakshi et al*.* 2019; Patel et al. 2020). Although such technologies sometimes achieve faster remediation, high operational costs and failure to achieve complete detoxification limit their applications (Zheng et al. 2020; Rong et al. 2021). Bioremediation has emerged as a cost-effective, sustainable, and efficient technology for cleaning up PAHs from contaminated environments (Kuppusamy et al. 2017; Patel et al. 2020). Bioaugmentation is a bioremediation strategy that involves the introduction of superior PAHs-degrading microbial inoculants of single strains, constructed consortia, or genetically engineered bacteria to contaminated sites for enhanced removal of the chemicals of concern (Megharaj et al. 2011; Tyagi et al. 2011; Lu et al. 2019a, b; Zheng et al. 2020; Rong et al. 2021; Martínez-Toledo et al. 2022). As a result of the long history of co-existence with contaminants, efficient PAHs degrading bacteria have frequently been found in legacy contaminated sites (El Fantroussi and Agathos 2005; Haritash and Kaushik 2009; Larsson et al. 2018; Cao et al. 2020). The classical approach to developing effective bacterial inoculants begins with selective enrichment, isolation, and screening of the best-performing strain(s) (Tyagi et al. 2011). Bacterial inoculants developed based on the screening and customized pre-adaptation process have been successfully implemented for PAHs bioremediation (Venkata Mohan et al. 2008; Silva et al. 2009; Sun et al. 2012; Kuppusamy et al. 2016b; Lu et al. 2019a, b; Chen et al. 2022). The classical approach relies on laboratory culture techniques for pure culture isolation and consortium construction. However, the reductionist approach may fall short in unraveling the PAHs-degrading potential of bacteria, especially when the organisms are not amenable for laboratory cultivation or cannot perform alone without support from other members of their communities.

For the selective enrichment of bacteria, PAHs are often supplemented to the liquid culture medium from concentrated stock solutions prepared in an organic solvent, such as dimethylformamide (DMF), dimethylsulfoxide (DMSO), hexane, or acetone. While the latter two highly volatile solvents are usually allowed to evaporate from the culture flask before adding culture medium and inoculum, DMF and DMSO are generally present in the culture suspension. Ironically, many of such supposedly inert organic solvents are biodegradable. For instance, several bacteria can efficiently mineralize DMF (Nisha et al. 2015; Zhou et al. 2018; Lu et al. 2019a, b) and DMSO (Murakami-Nitta et al. 2003; Hwang et al. 2007). Thus, the solvents may also act as co-substrates in enrichment microcosms and thereby manifest an effect on shaping the community structure.

To obtain an effective bioaugmentation inoculant, we initiated selective enrichment of PAHs-degrading bacteria from a former MGP site soil. The enriched culture degraded the target PAHs and the carrier solvent, DMF, and exhibited methylotrophic nutrition mode. Surprisingly, none of the pure strains could show PAHs degradation to a magnitude comparable to the source enrichment culture. In this study, we discuss the bacterial community structure of the cryptic enrichment culture, examine its ability to degrade the representative PAHs of increasing structural complexity level in a liquid medium, and finally demonstrate the suitability of the culture for bioremediation in a bench-scale slurry system.

## Materials and methods

### Soil samples

The MGP soil (MGPS) was collected at 0–20 cm depth from a long-term PAHs-contaminated former MGP site in Newcastle, New South Wales, Australia. The MGP site, operational for an extended period of about 70 years, was decommissioned in 1985. The aged MGPS used in the present study was a neutral sandy loam soil. The sample was temporarily stored at 4 °C before use. The laboratory waste soil (LWS) was initially collected from Highfields residential suburb of Newcastle, New South Wales, Australia. It was artificially spiked with phenanthrene, pyrene, and benzo(a)pyrene (BaP) in previous studies investigating PAHs' toxicity to the earthworm, *Eisenia fetida*. This soil, which would otherwise be discarded as contaminated soil waste after the investigations, was also used in bench-scale bioremediation experiments. The soil samples were air-dried and passed through a 2-mm stainless steel sieve. The pH and electrical conductivity (EC) were measured using a Mettler Toledo pH/Conductivity meter in soil: Milli-Q water (1:5, w/w) suspension after one hour of shaking. Sand, silt, and clay content were determined using the hydrometer method (Gee and Or 2002). Total carbon, nitrogen, and sulfur contents were measured in finely ground soil samples by dry combustion using a LECO TruMac CNS analyzer (630-300-400, LECO Corporation, Saint Joseph, Michigan, USA). The major physicochemical characteristics of the soils are provided in Table 1. The major and trace elements were extracted using the microwave-assisted aqua regia digestion method (USEPA method 3051A). Briefly, a 0.5 g soil sample was digested with 5 mL aqua regia (HNO_3_:HCl; 1:3) in a MARS 6 microwave digestion system. After cooling, the digest was passed through a 0.45 µm syringe-driven filter. Elemental analyses were performed in inductively coupled plasma mass spectrometry (ICP-MS, PerkinElmer NexION 350, USA) and inductively coupled plasma optical emission spectrometry (ICP-OES, PerkinElmer Avio 200, USA). The concentrations of heavy metals in both MGPS and LWS were well below the guideline values specified in the Australian National Environment Protection (Assessment of Site Contamination) Measure, Schedule B7 (NEPM 2013). PAHs were extracted following a modified ultrasound-assisted solvent extraction method (Subashchandrabose et al. 2017) and analyzed using gas chromatography-mass spectrometry (GC–MS) as detailed in the Supplementary Information. Based on the measured concentration of Σ16 PAHs (Table 1), the MGPS cannot be categorized as contaminated. However, as shown in Table 1, the soil contained significant amounts of four- and five-ringed PAHs. In addition, the BaP toxic equivalent quotient (BaP-TEQ) value exceeded all the Health-Based Investigation Levels (HILs) (NEPM 2013), suggesting that the carcinogenic PAHs in the MGPS can pose the health risks in humans. The LWS was contaminated with 163.80 mg kg^–1^ phenanthrene, 162.58 mg kg^–1^ pyrene and 21.56 mg kg^–1^ BaP (Table 1).

**Table 1:** Major physicochemical characteristics, elemental composition, and PAHs concentrations in the soils used in the study^a^

Characteristic/Element	Manufactured gas plant soil (MGPS)	Laboratory waste soil (LWS)
pH	7.7	6.7
EC	86.47	135.3
Sand (%)	73	20
Silt (%)	10	44
Clay (%)	17	36
Total carbon (%)	2.40	3.58
Total nitrogen (%)	0.07	0.29
Total sulfur (%)	0.08	0.04
Major elements (g kg^**–1**^)		
Mg	4.09	1.29
Al	11.72	6.61
P	0.04	0.73
S	0.08	0.08
K	2.60	1.20
Ca	17.33	2.71
Fe	13.32	10.44
Mn	0.15	0.17
Trace elements (mg kg^**–1**^)		
Cr	14.59	14.77
Co	5.36	4.08
Ni	5.66	6.51
Cu	12.53	15.00
Zn	55.40	75.29
As	4.49	3.76
Cd	0.11	0.34
Pb	46.16	23.31
2- and 3-Ringed PAHs (mg kg^–1^)		
Naphthalene	‒	–
Acenaphthylene	4.98	‒
Acenaphthene	‒	‒
Fluorene	1.21	‒
Phenanthrene	17.27	163.80
Anthracene	6.90	‒
Fluoranthene	40.99	‒
4-Ringed PAHs (mg kg^–1^)		
Pyrene	38.53	162.58
Benzo(a)anthracene	17.06	‒
Chrysene	16.13	‒
Benzo(b)fluoranthene	12.79	‒
Benzo(k)fluoranthene	12.61	‒
5- and 6-Ringed PAHs (mg kg^–1^)		
Benzo(a)pyrene	25.35	21.56
Dibenzo(a,h)anthracene	10.92	‒
Benzo(g,h,i)perylene	16.33	‒
Indeno(1,2,3-CD)pyrene	12.46	‒
Total PAHs (mg kg^**–1**^)	233.53	347.94
BaP-Toxic Equivalent Quotient (TEQ)^b^	42.09	21.56

^a^Mean values of triplicate samples

“‒”indicates the absence

^b^BaP-TEQ concentrations were calculated by multiplying the concentrations of each PAH compound with its individual toxic equivalence factor (TEF) for cancer potency relative to BaP. The TEF values of benzo(a)anthracene, benzo(b)fluoranthene, benzo(k)fluoranthene, BaP, benzo(g,h,i)perylene, chrysene, dibenz(a,h)anthracene and indeno(1,2,3-cd)pyrene as specified in NEPM (2013) were used to calculate BaP-TEQ in this study

### Enrichment and screening for PAHs-degrading pure cultures

Phenanthrene (99.5% purity), pyrene (99% purity), and BaP (≥ 96% purity) were purchased from Sigma-Aldrich. Individual stock solutions of these PAHs were prepared in DMF and acetone, stored at − 20 °C in amber glass vials, and thawed to room temperature before use. The enrichment culture was established by inoculating one-gram of MGPS into 50 mL M9 mineral medium and supplementing with 127.5 µL of 80 g L^–1^ DMF-dissolved pyrene stock solution to provide a final concentration of 200 mg L^‒1^. The M9 medium contained, per litre of water, 6.0 g Na_2_HPO_4_; 3.0 g KH_2_PO_4_; 1.0 g NH_4_Cl; 0.5 g NaCl; 0.2 g MgSO_4_·7H_2_O; 0.02 g CaCl_2_·2H_2_O; and 1 mL of non-chelated trace elements solution, SL-10 (Widdel et al*.* 1983). The detailed enrichment protocol and incubation conditions were described in our previous report (Dhar et al. 2020).

For the isolation of PAHs-degrading pure cultures, three strategies were followed. Aliquots of the enrichment culture from the 10th subculture were serially diluted and plated on (a) M9 agar supplemented with 100 mg L^–1^ DMF-dissolved phenanthrene or pyrene, (b) M9 agar with phenanthrene or pyrene crystals supplied in petri dish lids, and (c) 20% tryptic soy agar (TSA). Well-grown colonies were purified on their respective agar medium. The purified isolates were then examined for their ability to utilize phenanthrene and pyrene in a liquid culture medium. Briefly, the M9 medium was supplemented with 200 mg L^–1^ phenanthrene or pyrene from DMF-dissolved stock solution and inoculated with pure isolate to attain an initial OD_600 nm_ of 0.1. An additional culture set was also included where aliquots from acetone-dissolved phenanthrene or pyrene stock solution were placed at the bottom of the culture flask, and the M9 medium was added after the solvent evaporation. Culture showing increased turbidity during 14 days of incubation was further subcultured for three generations under the same conditions. Finally, PAHs from the triplicate liquid cultures were extracted and analyzed for confirmation of biodegradation.

### Methylotrophic substrate utilization and nitrogen metabolism

The growth of the enrichment culture on C1 compounds and other methylotrophic substrates was examined according to our previous protocol (Dhar et al. 2020). Briefly, the nitrogen-free (N-free) M9 medium was supplemented with 1000 mg L^–1^ methyl formamide, 1000 mg L^–1^ formamide, 500 mg L^–1^ methylamine, or 500 mg L^–1^ dimethylamine. Similarly, the M9 medium was supplemented with 1000 mg L^–1^ methanol, 500 mg L^–1^ formic acid, or 500 mg L^–1^ formaldehyde. All the culture flasks were inoculated with the mixed enrichment culture to attain an initial cell density of OD_600 nm_ of 0.1. The flasks were incubated on an orbital shaker with 125 rpm constant shaking at 25 ± 1 °C. After 72 h of incubation, cell density was measured from a 200 µL sample in a PerkinElmer EnSight™ multimode plate reader at 600 nm. Free-living N_2_-fixation, nitrate reduction and denitrification abilities were characterized according to the methods described previously (Dhar et al. 2020).

### DNA extraction, 16S rRNA gene sequencing, and bioinformatics analysis

Genomic DNA from the 20th generation enrichment culture, grown on 200 mg L^–1^ DMF-dissolved pyrene, was extracted in triplicate using DNeasy UltraClean Microbial Kit as per the manufacturer’s protocol. Bacterial diversity profiling was performed by sequencing 16S V1-V3 region amplicons on Illumina MiSeq platform. The sequences were analyzed using the Quantitative Insights into Microbial Ecology (QIIME 1.9.1) bioinformatics pipeline (Caporaso et al. 2010). The raw sequence reads of 16S V1-V3 region amplicons, obtained using triplicate gDNA templates of MM34X, have been submitted to NCBI Sequence Read Archive (SRA) under the BioProject accession number PRJNA804367 with the individual accession numbers: SAMN25733117, SAMN25733118, and SAMN25733119.

### Biodegradation of PAHs in liquid medium in the presence of DMF as co-substrate

The batch experiment was conducted to determine the ability of the mixed culture to degrade the three representatives of PAHs: phenanthrene, pyrene, and BaP. Inoculum for this experiment was developed by growing the mixed culture in 200 mL M9 medium in a 1 L Erlenmeyer flasks supplemented with 200 mg L^–1^ DMF-dissolved pyrene. Culture growing at mid-logarithmic phase was harvested by centrifugation at 4300 × *g* for 10 min at 4 °C, washed twice with M9 medium, and resuspended in the same medium to obtain a dense cell suspension having OD_600 nm_ of 1.0. The batch biodegradation experiment was carried out in 10 mL total culture volume contained in sterile 40 mL amber glass vials sealed with PTFE-lined screw caps. An appropriate amount of DMF-dissolved stock solution of phenanthrene (100 g L^–1^), pyrene (100 g L^–1^) or BaP (12.5 g L^–1^) was added to the corresponding vials to provide a final concentration of 200 mg L^–1^ phenanthrene, 200 mg L^–1^ pyrene or 25 mg L^–1^ BaP. The addition of PAHs solution from the stocks also provided ~ 2000 mg L^–1^ DMF to the culture medium. All treatments were inoculated with the cell suspension to provide an initial cell density OD_600 nm_ of 0.1. Parallel vials containing medium and PAH solution, but no added inoculum served as the controls for assessing abiotic loss. The vials were incubated on an orbital shaker with constant shaking at 125 rpm in a 25 ± 1 °C dark room. Triplicate vials from the incubation set were withdrawn at specified intervals for liquid–liquid extraction of the PAHs. Residual PAHs were analyzed using an HPLC system equipped with a fluorescence detector (FLD). Detailed PAHs extraction protocol and HPLC-FLD operating parameters are provided in the Supplementary Information.

A separate set was incubated with 200 mg L^–1^ pyrene, and aliquots were periodically withdrawn for residual DMF analysis by HPLC according to the method described previously (Dhar et al. 2020).

### Utilization of PAHs as sole carbon and energy sources

The enrichment culture’s ability to utilize the PAHs as the sole carbon and energy source was examined. Aliquots of the acetone-dissolved PAHs stock solutions were placed at the bottom of sterile 40 mL amber glass vials to provide 200 mg L^–1^ phenanthrene, 200 mg L^–1^ pyrene, or 25 mg L^–1^ BaP. After the evaporation of acetone and the appearance of a thin PAH crystal layer, the M9 medium was added immediately to minimize the strong adherence of the crystal to the vials’ wall. The suspension was then inoculated with culture to provide an initial cell density OD_600 nm_ of 0.1. Control vials containing M9 medium and PAH crystal with no added inoculum served as controls. In each case, three independent replicates were included. In addition, a parallel set of inoculated culture medium was supplemented with DMF-dissolved PAHs to examine the effect of DMF as the co-substrate. All the vials were incubated on an orbital shaker with constant shaking at 125 rpm in a 25 ± 1 °C dark incubation room. Triplicate vials were withdrawn at a specified sampling time for the extraction and subsequent quantification of residual PAHs by using an HPLC-FLD system as outlined in the Supplementary Information. Total cellular protein was determined according to the Bradford method as described elsewhere (Dhar et al. 2020).

### PAHs biodegradation in slurries of contaminated soils

The efficiency of the enrichment culture in the bioremediation of PAHs from MGPS and LWS was evaluated in a laboratory bench-scale 1:2 (w/v) soil:water slurry system. The experiment was conducted in 40 mL amber vials fitted with a screw cap and PTFE-lined septa. Each treatment vial received 5-g air-dried soil, 9 mL distilled water, and 1.0 mL culture suspension. The culture suspension was obtained by washing the cell pellet harvested from a mid-logarithmic phase culture grown on 200 mg L^–1^ pyrene supplied from DMF stock and resuspending in sterile water to obtain an OD_600 nm_ of 1.0. The contents in the vials were mixed thoroughly and incubated in dark (to prevent photodegradation of the PAHs) at 25 ± 1 °C on an orbital shaker with 125 rpm constant shaking. A parallel set of vials with the same amount of soil and 10 mL water, but no inoculum, was also maintained to assess the removal of PAHs by natural attenuation. A third uninoculated slurry set was kept in a − 20 °C freezer to assess any abiotic loss. Triplicate vials were withdrawn periodically for PAHs extraction and analysis. Residual PAHs from the soil slurry were extracted following the modified ultrasound-assisted extraction method (Subashchandrabose et al. 2017) using acetone-hexane (1:1, v/v) as the solvent. PAHs analysis was carried out with an Agilent 7890B gas chromatograph equipped with an Agilent 7693A automatic liquid sampler and an HP-5MS capillary column (30 m, 0.25 mm i.d., 0.25 μm film thickness), coupled with an Agilent 7000A triple quadrupole mass spectrometer operating in electron impact ionization mode. All the measurements were expressed on a soil dry-weight basis. The detailed extraction procedure, GC–MS operating conditions, and quality control measures are provided in the Supplementary Information.

### Statistical analysis

All statistical analyses and data plotting were performed using OriginPro 2021 (OriginLab Corporation, Northampton, MA, USA). The significance of the difference between the residual PAHs in treatments with or without DMF was determined by a two-tailed independent *t*-test, assuming DMF might have a positive or a negative effect. Statistical significance of the differences among the residual PAHs in slurry phase bioremediation experiments were calculated by two-way factorial ANOVA, considering the outcomes were dependent on culture conditions (abiotic *vs* natural attenuation *vs* bioaugmentation) and incubation time (0, 7, 14, and 28 days) at 95% confidence interval followed by Tukey’s multiple comparison test.

## Results and discussion

### Enrichment of the methylotrophic PAHs-degrading culture

In this study, the selective enrichment effort was aimed at the development of an efficient PAHs-degrading bacterial bioaugmentation inoculant. The initial enrichment was established by inoculating the MGPS into the mineral medium supplemented with DMF-dissolved pyrene to serve as the sole carbon and energy source. After repeated subculturing, it became evident that the enrichment culture, designated as MM34X, utilized the supplied PAH and, unexpectedly, the carrier solvent, DMF. Previously we reported DMF degradation by *Mesorhizobium tamadayense* MM3441 isolated from the enrichment culture (Dhar et al. 2020). DMF utilization by the enrichment culture led to the speculation that the culture could grow on other methylotrophic substrates. MM34X rapidly utilized several C1 (formic acid, formaldehyde, formamide, methanol and methylamine) and methylotrophic multi-carbon substrates (methylformamide and dimethylamine) (Table 2). The culture also exhibited free-living N_2_ fixation and denitrification abilities.

**Table 2:** C1 and multi-carbon methylotrophic substrate utilization and nitrogen metabolism ability of the enrichment culture MM34X

Growth on methylotrophic substrates^a^	
Formic acid	+
Formaldehyde	+
Formamide	+
Methanol	+
Methylamine	+
Methylformamide	+
Dimethylamine	+
Nitrogen metabolism	
Nitrogen fixation^b^	+
NO_3_^–^ to NO_2_^–^	+
NO_2_^–^ reduction	+

^a^“ + ” indicates an increase in the medium turbidity by at least 0.5 OD_600 nm_ compared to the substrate-free control after 36 h of incubation

^b^“ + ” indicates an increase in the medium turbidity by 0.3 OD_600 nm_ when grown on glucose in the N-free M9 medium + 3.5 mM molybdate compared to the glucose in N-free M9 medium without molybdate, and substrate-free control in N-free medium + 3.5 mM molybdate after 4 days of incubation

Four distinct pure bacterial isolates were obtained when the MM34X was plated on the M9 agar medium supplemented with phenanthrene or pyrene in the presence or absence of DMF co-substrate. Among the isolates, only *M. tamadayense* MM3441 degraded phenanthrene and pyrene, both in the presence or absence of DMF, but at a significantly slower rate compared to the culture MM34X. The smaller number of pure isolates obtained from the solid agar surfaces, comparatively slow degradation of the PAHs by MM3441, and failure of the rest of the pure cultures to degrade the PAHs suggested that the predominant PAH-degrading organism(s) in the MM34X probably could not grow on agar medium. The apparent inability can be attributed to the lack of essential growth factors (e.g., vitamins) in the mineral salts medium. Since TSA medium consists of many critical growth factors, a culture aliquot was placed on 20% TSA medium to allow the growth of fastidious bacteria. Although more distinct colonies (*n* = 6) were obtained on the TSA agar surface, not a single isolate showed appreciable PAH degradation ability compared to the mixed enrichment culture.

### Bacterial community structure in the enrichment culture

**Fig. 1:**
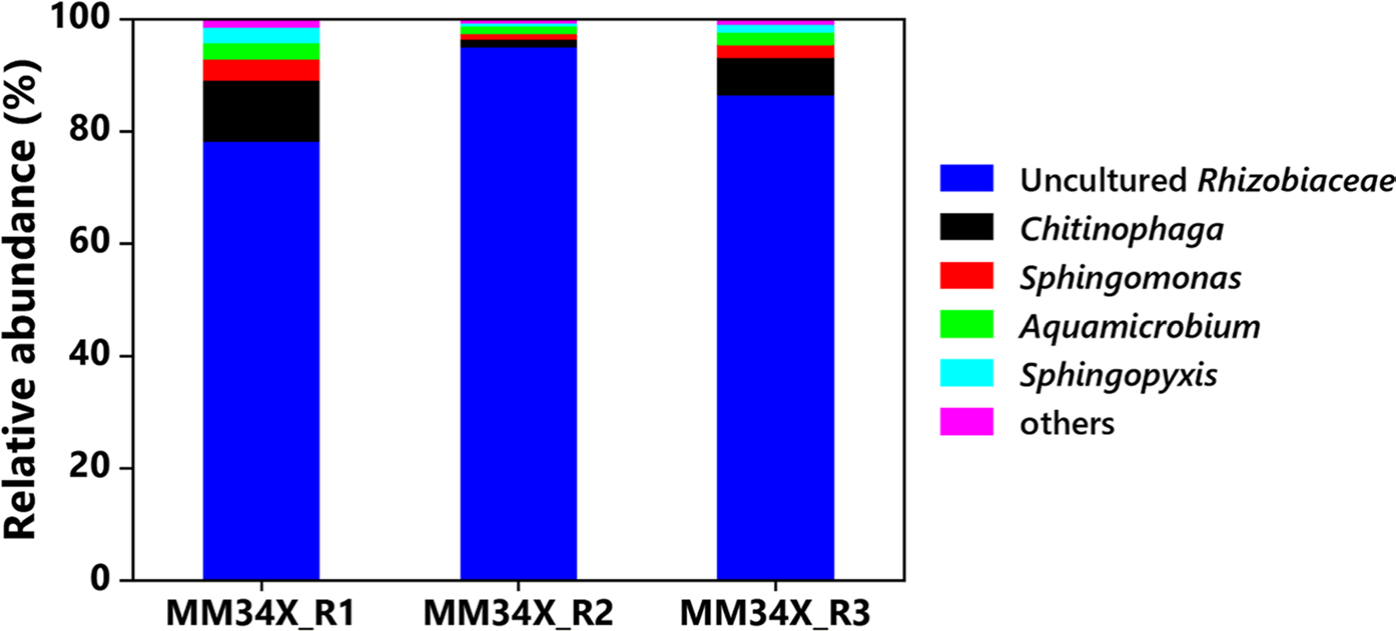
Relative abundance (%) of bacterial taxa at the genus level in the enrichment MM34X. The bacterial diversity profiling was conducted in triplicates; MM34X_R1, MM34X_R2 and MM34X_R3 represent independent biological replicates. Taxa observed at ≤ 0.4% relative abundance are combined into the "Others" category

To get a stable bacterial community, the enrichment MM34X was periodically subcultured for twenty generations under the same culture conditions. Moreover, the 16S rRNA amplicon sequencing was performed on triplicate samples to account for PCR and sequencing biases. The rarefaction curves for the replicates were approximately levelled off, and they differed slightly from each other (data not shown), suggesting that the sequencing depth was satisfactory enough to capture most of the species. The bacterial community structures at the genus level in all the replicates are shown in Fig. 1. Surprisingly, the MM34X community was found to be dominated (86.6 ± 8.4%) by a previously uncultured member of the family *Rhizobiaceae*. An attempt has been made to assign genus-level taxonomic affiliation to the dominant OTU by searching similarities in the SILVA, RDP, GTDB, LTP, and EMBL–EBI/ENA databases. However, even at a minimum 90% similarity threshold, the OTU did not match with any known genus. The dominance of the uncultured *Rhizobiaceae* bacterium in the highly enriched MM34X community suggests its active functional role in the community. As a result, the bacterium cannot be easily fitted under the “unculturable” (Stewart 2012) or “viable but not culturable (VBNC)” (Oliver 2005) class of bacteria. The finding highlights the importance of paying attention to the mixed bacterial culture rather than focusing only on monoculture or constructed consortium during the development of inoculants for bioremediation. Other bacterial genera that constituted minor fractions of the community were *Chitinophaga* (6.3 ± 4.8%), *Sphingomonas* (3.8 ± 1.4%) *Aquamicrobium* (2.2 ± 0.7%), and *Sphingopyxis* (1.5 ± 1.2%), while *Mesorhizobium* represented only 0.2 ± 0.03% of the entire community (Fig. 1). Strains of the genus *Sphingomonas* are well-established PAHs degraders (Pinyakong et al. 2003). The genus *Chitinophaga* comprises strains of chitinolytic, filamentous and gliding bacteria (Sangkhobol and Skerman 1981) and has been occasionally reported to get enriched in the soil during PAHs bioremediation (Aburto-Medina et al. 2012; Subashchandrabose et al. 2019). Similarly, the involvement of *Aquamicrobium* sp. in PAHs degradation has been rarely reported (Andreoni et al. 2004). However, it is difficult to predict any active role of the genera in the enrichment of MM34X.

Members of *Rhizobiaceae* are distributed among diverse habitats; a significant proportion of them adapts symbiotic lifestyle with hosts (Sachs et al. 2011) and often colonizes at rhizosphere region (Hayat et al. 2010). Several members of the family *Rhizobiaceae,* such as *Rhizobium*, *Sinorhizobium,* and *Agrobacterium*, can degrade PAHs (Keum et al. 2006; Yessica et al. 2013; Kuppusamy et al. 2016a). Availability of nitrogen is critical in determining the success of field-scale bioremediation of PAHs. Thus, the culture MM34X is expected to would succeed in overcoming nitrogen limitations in contaminated soils.

The culture MM34X can adopt methylotrophic nutrition mode as evidenced by its ability to grow on C1 compounds, DMF, and other methylotrophic substrates (Table 2). Methylotrophs are ubiquitous inhabitants of soil, sediments, and freshwater (Anthony 1982; McDonald et al. 2001; De Marco et al. 2004). While numerous methylotrophs from diverse taxonomic classes have already been identified, many remain uncultured (McDonald and Murrell 1997; Radajewski et al. 2000, 2002). The application of methylotrophic bacteria for sustainable agriculture (Kumar et al. 2016) and industrial biotechnology (Trotsenko et al. 2005; Schrader et al. 2009) are well known. However, their role in the bioremediation of pollutants remains underappreciated. The current understanding of methylotrophs and their function in carbon metabolism is updating. Many noble and phylogenetically diverse members with previously unknown functional roles have been identified (Chistoserdova et al. 2009; Chistoserdova 2011; Chen 2012; Wischer et al. 2015). For example, *M. tamadayense* MM3441, isolated from the MM34X enrichment, was reported as the only second member of Rhizobiales involved in methylotrophy (Dhar et al. 2020). Accordingly, the current findings demonstrate the role of methylotrophic mixed culture in organic pollutants degradation.

### Biodegradation of PAHs in the liquid medium

**Fig. 2:**
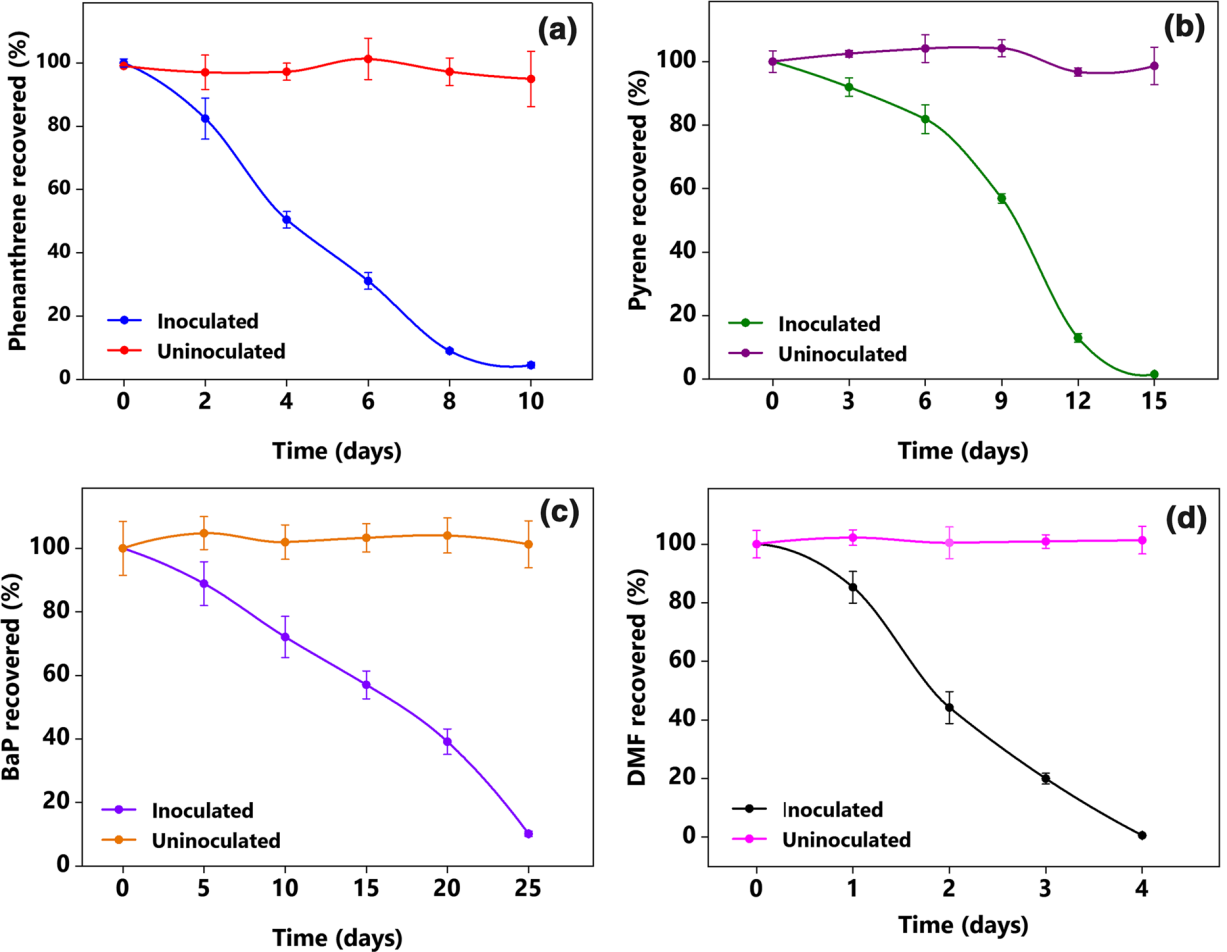
Biodegradation of the PAHs and the co-substrate DMF by MM34X in liquid medium: **a** 200 mg L^–1^ phenanthrene, **b** 200 mg L^–1^ pyrene, **c** 25 mg L^–1^ BaP, all in the presence of DMF, and **d** 2000 mg L^–1^ DMF. Data represent mean ± standard deviation (SD) (*n* = 3)

PAHs with different molecular structures are formed during the incomplete combustion of fossil fuels and biomass. Thus, MGP sites contain a variety of PAHs mixture rather than a single compound. Therefore, evaluating the ability of a candidate inoculant in the biodegradation of representatives PAHs from the structural groups is necessary before proceeding to a laboratory bench-scale feasibility study. In this study, phenanthrene, pyrene, and BaP were selected as the representatives of three-, four-, and five-ringed PAHs. The biodegradation of 200 mg L^–1^ of phenanthrene, 200 mg L^–1^ of pyrene, and 25 mg L^–1^ of BaP by MM34X were evaluated in the liquid medium in the presence of 2000 mg L^–1^ DMF as a co-substrate (Fig. 2). The culture completely degraded the supplied phenanthrene in 10 days of incubation. Exponential degradation commenced on the second day of incubation and continued till the eighth day; during this period, almost 75% of the total phenanthrene was degraded at 0.37 day^–1^ (Fig. 2a). Pyrene was also completely removed from the medium but at a slower rate compared to phenanthrene. Almost 80% of the total pyrene was degraded during the exponential degradation phase at a rate of 0.21 day^–1^, and complete degradation occurred within the 15 days (Fig. 2b). The culture MM34X could also degrade the highly recalcitrant BaP, albeit at a significantly slower rate. While growing on 25 mg L^–1^ BaP in the presence of DMF, the culture removed 90% of the compound within 25 days of incubation (Fig. 2c). The calculated biodegradation half-life of BaP was as high as 6.4 days, compared to 3.2 and 1.9 days for pyrene and phenanthrene, respectively.

**Fig. 3:**
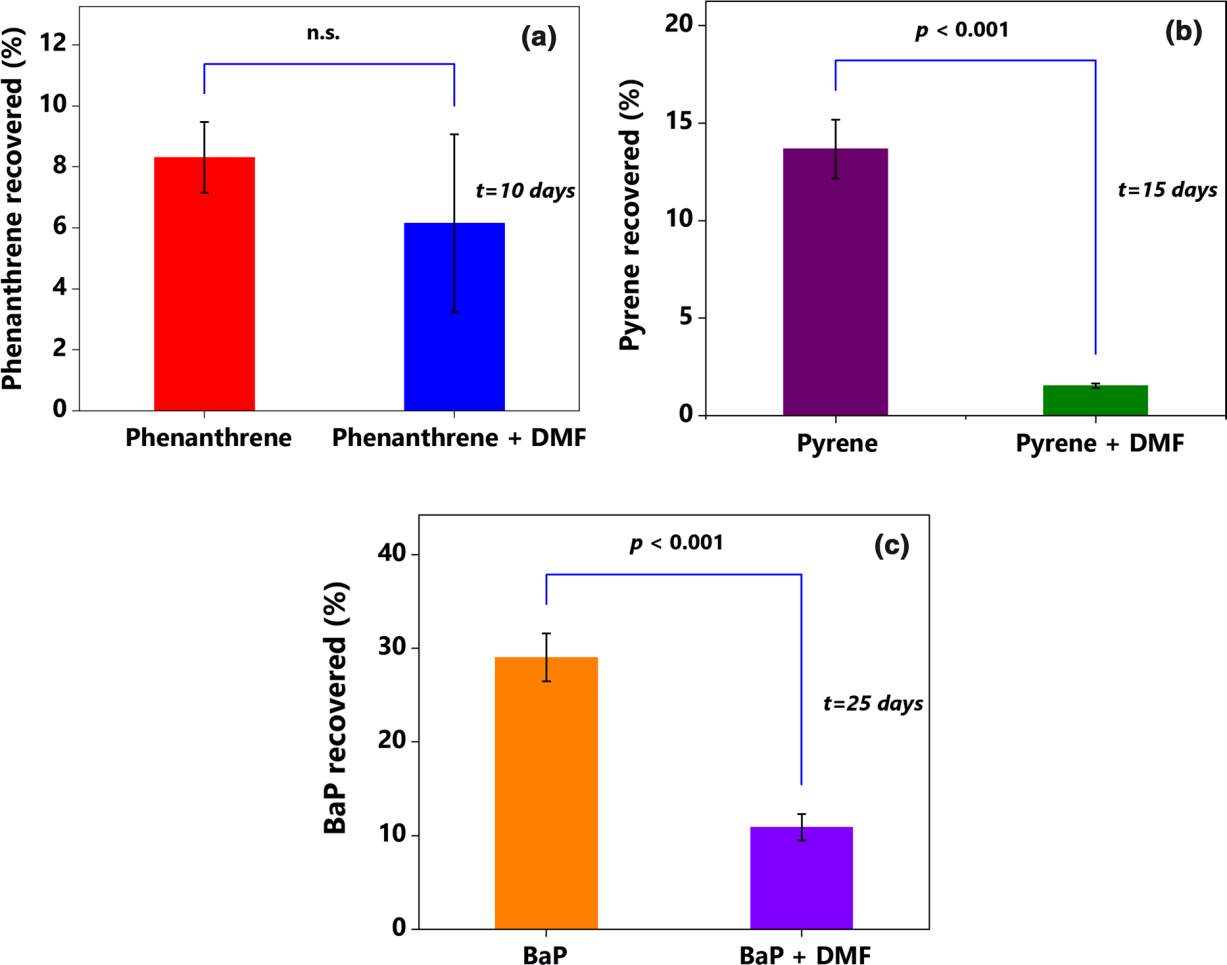
Biodegradation of **a** phenanthrene, **b** pyrene, and **c** BaP by MM34X when supplied as the sole carbon source and in the presence of 2000 mg L^–1^ DMF as the co-substrate. Columns represent mean ± SD (*n* = 3). *P* values are indicated on the top of bracket. *n.s.* not significant, *t* incubation time

During the degradation process, DMF was preferentially utilized over the PAHs. When the culture was supplemented with pyrene and DMF, complete removal of 2000 mg L^–1^ of the co-substrate was achieved within four days of incubation (Fig. 2d). Removal of approximately 40 mg L^–1^ pyrene (Fig. 2b) during the same period suggests the preferential utilization of the simpler carbon source from the medium. Simultaneous utilization of DMF and PAHs gave rise to the concerns of whether and to what extent the culture MM34X could utilize the selected PAHs as sole carbon and energy sources. To resolve the issues, MM34X was further examined for its ability to utilize the PAHs as the sole carbon and energy source in the absence of co-substrate. As shown in Fig. 3, the culture could degrade phenanthrene, pyrene, and BaP as the sole carbon sources without any co-substrate. Not only were the PAHs degraded, but the substrate-derived carbon was utilized for cellular biosynthesis, as indicated by the increase in total cellular protein content (Table 3). The presence of DMF as the co-substrate did not significantly (*P* < 0.05) alter the complete phenanthrene degradation (Fig. 3a); however, substantially higher degradation of pyrene and BaP was found in the presence of the co-substrate (Fig. 3b, c). In addition, BaP degradation in the absence of DMF was more affected than pyrene degradation (Fig. 3c). These observations have been significant because utilization of the contaminant in the absence of any co-substrate extends the potential of the culture for its use as a bioremediation agent. In the natural environment, PAHs generally co-occur with several pollutants such as metals, cyanides, and other aromatic compounds but not DMF. Therefore, any obligate dependency on DMF could severely limit the culture for the intended environmental application. The presence of DMF would either have a positive effect due to its role in better dispersion of the PAHs in the medium and as a growth enhancer or a negative effect due to its preferential utilization. The data presented in Fig. 3 contradict any adverse impact and instead establish the stimulatory role of DMF in the degradation of pyrene and BaP. Interestingly, when DMF was supplied as the sole carbon source, pyrene and BaP degradation were significantly affected. Higher resonance energy and very low solubility of pyrene and BaP in an aqueous medium and their tendency to stick to the glass wall could be the justifications behind the observed significantly (*P* < 0.001) less degradation.

**Table 3:** Utilization of the PAHs as the sole sources of carbon and energy

PAH	Incubation time (days)	Substrate utilized (mg L^–1^)^a^	Total cellular protein (mg L^–1^)^a,b^
Phenanthrene	10	183.4	39.2
Pyrene	15	172.6	37.9
BaP	25	17.75	8.8

^a^Means of three independent experiments

^b^Increase in total cellular protein from an initial value of 5 mg L^–1^

### Bioremediation of the PAHs-contaminated soils

MM34X was enriched and maintained in the liquid medium. In addition, the PAHs biodegradation potential has already been demonstrated in the liquid medium. However, conditions in contaminated environments (such as soil) are different from those in laboratory culture medium. Several biotic and abiotic factors determine the fate of PAHs in contaminated environments. PAHs are sparsely soluble in water and tend to sorb to soil minerals and organic matters rendering the pollutants less bioavailable for microbial uptake and degradation. As a result, their biodegradation becomes severely limited (Megharaj et al. 2011; Dhar et al. 2019). The survival and performance of an inoculant in contaminated environments determine the success of a bioaugmentation program. Therefore, bench-scale bioremediation experiments were carried out in a soil-slurry system with the MGPS and LWS to examine the performance of the culture.

**Fig. 4:**
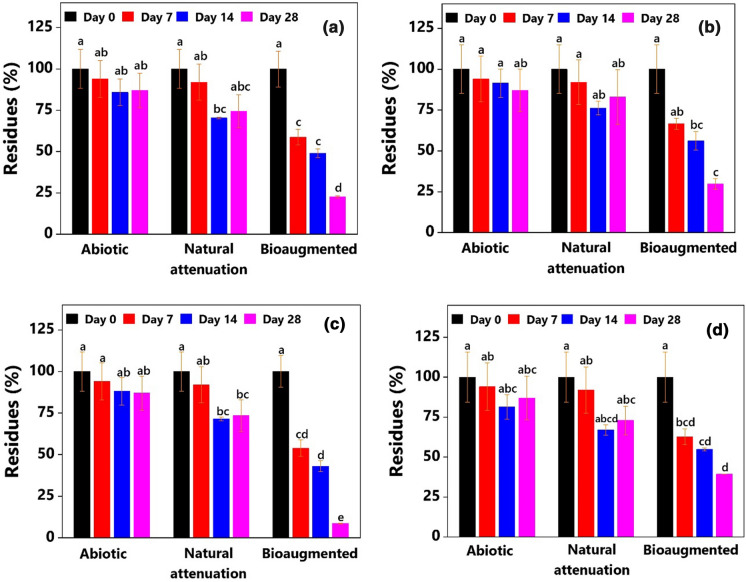
Enhanced removal of **a** USEPA Σ16 PAHs, **b** 2- and 3-ringed PAHs, **c** 4-ringed PAHs, and **d** 5- and 6-ringed PAHs in the bioaugmented MGP soil. The experiment was conducted in bench-scale soil: water (1:2, w/v) slurry. Columns represent mean ± SD (*n* = 3). Columns sharing the same letter are not statistically (*P* ˂0.05) different

The data in Fig. 4 show the success of inoculating the MGPS with the MM34X in removing the USEPA 16 PAHs over 28 days. Bioaugmentation with the MM34X had resulted in the significant removal of Σ16 PAHs (Fig. 4a) and constituting 2 to 3-ringed members (Fig. 4b), 4-ringed members (Fig. 4c), and the heavier 5 to 6-ringed members (Fig. 4d). During the incubation period, Σ16 PAHs were gradually decreased from the inoculated soils. After 28 days, almost 80% of Σ16 PAHs were removed by the culture. Natural attenuation accounted for only a 25% reduction during the same period (Fig. 4a). Two- to three-ringed PAHs constituted only 13% of the total initial PAHs in MGPS. Although natural attenuation resulted in approximately 20% reduction in 2 to 3-ringed PAHs from the soil, it was far less efficient compared to the inoculated treatment. Within the first seven days of incubation, almost 35% of 2 and 3-ringed PAHs were removed, whereas the maximum removal was nearly 70% on day 28 (Fig. 4b). A significant fraction (48%) of total PAHs in MGPS was contributed by 4-ringed members (Table 1). The culture efficiently removed more than 90% of the four-ringed PAHs. Again, natural attenuation fell short of removing more than one-fourth of the four-ringed constituents (Fig. 4c). The fraction constituted by five- and six-ringed members is of particular interest due to their complex ring structure, minimal solubility, high resistance to degradation, and carcinogenic properties. Despite their recalcitrance, inoculation of the MGPS with MM34X significantly facilitated the removal of the fraction (Fig. 4d). Almost 60% of 5- and 6-ringed PAHs were degraded in inoculated soil samples after 28 days of incubation, while natural attenuation accounted for 30% of removal (Fig. 4d). Under the natural attenuation process, the amounts of all the PAHs removed during the last 14 days (day 14–28) were minimal (Fig. 4). This observation may be explained assuming the depletion of the bioavailable fraction.

**Fig. 5:**
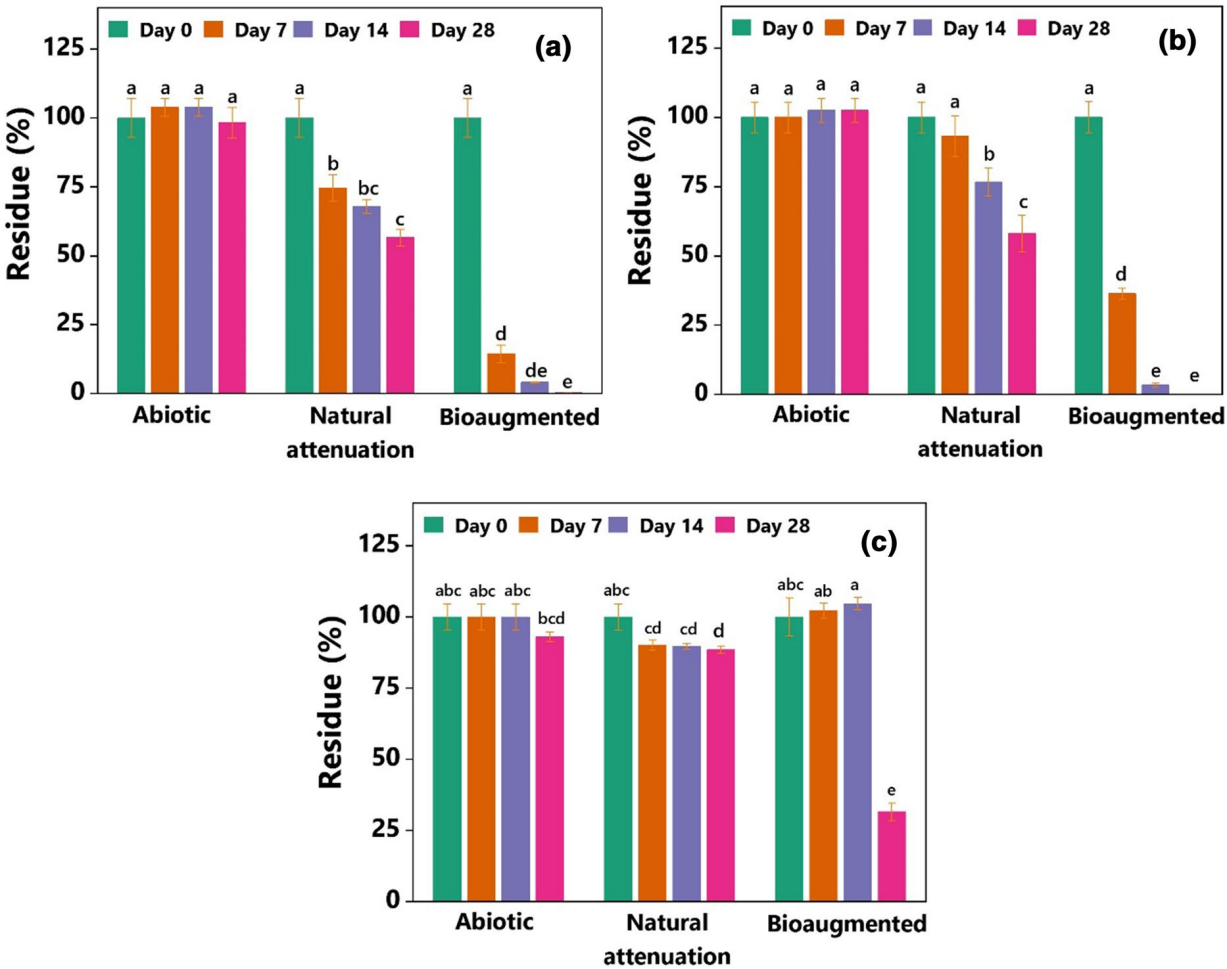
Enhanced removal of **a** phenanthrene, **b** pyrene, and **c** BaP in the bioaugmented laboratory waste soil (LWS). The experiment was conducted in bench-scale soil: water (1:2, w/v) slurry. Columns represent mean ± SD (*n* = 3). Columns sharing the same letter are not statistically (*P* ˂ 0.05) different

Many laboratory ecotoxicology experiments require artificial spiking of soils with PAHs. After experimentation, such soils are generally disposed of as laboratory waste that costs a considerable amount for subsequent processing steps. The LWS was initially collected from a residential suburban area and artificially spiked with varying concentrations of phenanthrene, pyrene, and BaP for laboratory studies. The current study made an effort to decontaminate the LWS by inoculating with the MM34X. Since the culture MM34X was initially isolated from the MGPS, testing its efficiency in PAHs-contaminated LWS also provided the avenue for bioremediation assessment beyond the native soil type. The bioaugmentation approach had been largely successful for LWS (Fig. 5). Both phenanthrene and pyrene from the inoculated soil samples were removed entirely during incubation. Within the first seven days of incubation, almost 85% of the initial phenanthrene was removed (Fig. 5a), and the residual amount was reduced to only 4% after 14 days of incubation. Under the natural attenuation process, 45% removal of phenanthrene was observed, albeit at significantly lower efficiency compared to the inoculated soil samples. A similar trend was also observed in the case of pyrene. Nearly, 65% of the initial pyrene was removed just within seven days of incubation, and after 14 days, a negligible amount of pyrene residue was left in the inoculated samples (Fig. 5b). However, the natural attenuation process accounted for 40% removal of the initial pyrene from LWS. Therefore, it could be assumed that if allowed to proceed for a prolonged period, the natural attenuation process might also achieve the same level of remediation. However, not all PAHs are susceptible to the natural attenuation process. As shown in Fig. 5c, the residual BaP in soil was only removed by as much as 12% by the natural attenuation process. Interestingly, BaP concentration in the inoculated soil samples remained almost the same during the first 14 days of incubation. The residue of BaP fell below 35% at the end of the incubation (Fig. 5c). It should be mentioned that the LWS received organic fertilizer during its use in the earlier ecotoxicology experiments. Therefore, the available organic amendment might have influenced appreciable degradation by natural attenuation and faster outcomes in inoculated samples. The LWS was amended with cow dung during the ecotoxicology experiments to facilitate the growth of *E*. *fetida*. Previous reports suggest that a combined bioaugmentation-biostimulation strategy has been shown to achieve faster removal (Straube et al. 2003; Sun et al. 2012; Zeneli et al. 2019). Therefore, the organic amendment might have influenced the appreciable PAHs degradation under natural attenuation conditions and the faster outcomes in the bioaugmented treatments. As shown in Table 1, the aged MGPS was low in total C and N compared to the LWS. Hence, a combination of bioaugmentation and biostimulation with an organic or inorganic amendment may support quicker and more efficient bioremediation of the MGPS.

Most of the published reports described biodegradation and bioremediation of PAHs by single bacterial strains or defined constructed consortia. In numerous instances, multicomponent formulations have been found to be more efficient in PAHs degradation (Yu et al. 2005; Li et al. 2008; Kuppusamy et al. 2016b). Notably, several culture-independent DNA-stable isotope probe (DNA-SIP) based investigations have identified PAHs-degrading uncultured bacteria in different environments (Singleton et al. 2006; Gutierrez et al. 2011, 2013; Jones et al. 2011; Thompson et al. 2017; Thomas et al. 2019). However, PAHs biodegradation by mixed cultures consisting of functional uncultured bacteria has rarely been investigated. Dastgheib et al. (2012) reported the inability of *Halomonas* sp. isolated from a phenanthrene-degrading consortium and postulated that the other constituent, an uncultured *Marinobacter* sp., initiated the PAH degradation. The MM34X culture is a unique example showing an uncultured bacterium's dominance in a highly enriched laboratory culture. In addition, the findings also demonstrated the potential application of the enrichment culture, which was dominated by an uncultured bacterium, for the bioremediation of contaminated soils. Further genomic and metabolic investigations will be required to characterize the uncultured bacterium.

Heavier (≥ 5-ringed) PAHs constitute the most recalcitrant and carcinogenic fraction in a PAHs mixture. Therefore, bacterial inoculants with the ability to degrade both low and high molecular weight PAHs are desirable for treatment purposes. However, only a few bacteria can degrade BaP and other complex PAHs as the sole carbon source. Accordingly, in bacterial bioremediation experiments, removal of the recalcitrant fraction has been found to be the least efficient (Juhasz and Naidu 2000; Moody et al. 2005). The observations from the bench-scale feasibility testing experiments indicated that the MM34X degraded a significant amount of five- and six-ringed PAHs from the MGPS and BaP from the LWS. Therefore, the overall findings of the study established the potential of the culture for the bioremediation of the MGP site. In addition, applying the culture for the decontamination of laboratory waste would lead to the development of an efficient, economical, and in-house management technique.

## Conclusion

The present study demonstrates that a previously uncultured bacterium dominated in a PAHs-degrading bacterial enrichment culture. The mixed culture was more efficient in PAH degradation than its culturable constituents. Efficient removal of PAHs from a former MGP site and a laboratory waste soil indicated the suitability of the culture as a promising bioaugmentation formulation. Future investigations will be directed to evaluating field-scale performance and remediation action planning.

## Supplementary Information

Below is the link to the electronic supplementary material.

Supplementary file1 (DOCX 26 kb)
