# Supplementary material for: Efficient bioremediation of PAHs-contaminated soils by a methylotrophic enrichment culture
Source: Biodegradation. 2022 Aug 17;33(6):575–91. doi: 10.1007/s10532-022-09996-9 (PMC9581816; doi:10.1007/s10532-022-09996-9)
Supplement: Supplementary file 1 — Supplementary file1 (DOCX 26 kb) [file 10532_2022_9996_MOESM1_ESM.docx]

**Supplementary Information**

**Efficient bioremediation of PAHs-contaminated soils by a methylotrophic enrichment culture**

**Kartik Dhar^1^, Logeshwaran Panneerselvan^1,2^, Kadiyala Venkateswarlu^3^, Mallavarapu Megharaj^1,2^**^*^

^1^ Global Centre for Environmental Remediation (GCER), College of Engineering, Science and Environment, The University of Newcastle, ATC Building, University Drive, Callaghan, NSW 2308, Australia

^2^ Cooperative Research Centre for Contamination Assessment and Remediation of the Environment (CRC CARE), The University of Newcastle, ATC Building, Callaghan, NSW, 2308, Australia

^3^ Formerly Department of Microbiology, Sri Krishnadevaraya University, Anantapuramu 515003, India

****Address for correspondence:***

**Prof. Mallavarapu Megharaj**

Global Centre for Environmental Remediation (GCER)

College of Engineering, Science and Environment

The University of Newcastle, ATC Building

University Drive, Callaghan, NSW 2308, Australia

Mobile: +61411126857

E-mail: [megh.mallavarapu@newcastle.edu.au](mailto:megh.mallavarapu@newcastle.edu.au)

***Genomic DNA extraction, amplicon sequencing and bioinformatics analysis***

Genomic DNA from the mixed enrichment culture was extracted in triplicate using DNeasy UltraClean Microbial Kit as per the manufacturer's protocol. Microbial diversity profiling based on bacterial 16S rRNA gene was performed at the Australian Genome Research Facility (AGRF) (Melbourne, Australia). Amplicons corresponding to the 16S V1-V3 region, generated using the primer set 27F (5′–AGAGTTTGATCMTGGCTCAG–3′) and 519R (5′–GWATTACCGCGGCKGCTG–3′), were sequenced on the Illumina MiSeq platform, utilizing Illumina’s Nextera XT Indexes and Paired End sequencing chemistry. The demultiplexed paired-end sequences were analyzed using the Quantitative Insights into Microbial Ecology (QIIME 1.9.1) bioinformatics pipeline (Caporaso *et al.* 2010). Chimeric sequences were removed using USEARCH 6 (Edgar 2010) with *de novo* model. Sequences were clustered against 99% reference data sets of Silva (v132 release) databases (Quast *et al.* 2013). Sequences that did not match any entries were subsequently clustered into open reference, where reads were clustered against a reference sequence collection. Any reads that did not hit the reference collection were clustered *de novo*.

***Extraction and analysis of PAHs from liquid medium***

Residual PAHs in the culture supernatant was extracted by ultrasound-assisted liquid-liquid extraction according to the method described by Subashchandrabose *et al*. (2017) with some modifications. PAHs are hydrophobic, do not distribute evenly in culture supernatant and often adhere to the glass culture vials. To ensure the correct estimation, the whole culture liquid medium was sacrificed for extraction. An equal volume of ethyl acetate was added to the culture medium, vortexed vigorously for a min and then the organic residues were extracted by ultrasonication. The ultrasonication was performed in a 360 W ultrasonic water bath (Ultrasonics Australia) with a mean operating frequency of 40 Hz for 15 min. The extraction was repeated twice, and the organic extracts were pooled. One mL extract was passed through a syringe-driven 0.22 µm PTFE membrane filter and stored in a 2 mL amber GC vial at –20 °C before analysis. The samples were diluted with ethyl acetate before injection. The extracts were analyzed in an Agilent 1260 Infinity II HPLC system equipped with an Agilent G7121B Spectra series multi-signal fluorescence detector (FLD). A sample volume of 10 µL was injected at 25 °C and PAHs were separated on an Agilent ZORBAX Eclipse Plus C18 column (4.6 × 150 mm, 3.5 μm particle size) at 1 mL min^–1^ flow rate. Methanol and water were used as the mobile phase: 80% methanol between 0 and 11 min, and a linear gradient from 80 to 100% methanol between 11 and 13 min, 100% methanol from 13 to 18 min. The total rum time was 21 min, including 3 min post-run.

PAHs were detected with FLD at optimized excitation and emission wavelengths for individual PAHs. The λ_excitation (nm)_/λ_emission (nm)_ for phenanthrene, pyrene, and BaP were 250/364, 250/388, and 250/410, respectively. The limits of detection for phenanthrene, pyrene and BaP were 25, 25, and 10 ng mL^‒1^, respectively. PAHs concentration in the samples was determined from seven points external calibration curves obtained with certified reference solution for phenanthrene (Sigma-Aldrich, Supelco, 40079), pyrene (Sigma-Aldrich, Supelco, 40082) and BaP (Sigma-Aldrich, Supelco, CRM40071). The extraction efficiency was evaluated based on the recovery of a known quantity of spiked analytes from the M9 medium. The stability, precision and accuracy of the chromatographic procedure were evaluated by monitoring the concentration of a calibration standard injected after every ten samples. To avoid overestimation of PAHs due to carryover, ethyl acetate solvent blank was injected after every 10 samples. The extraction efficiencies were 96±10 % (n=6) for phenanthrene, 98±12 % (n=6) for pyrene, and 103±15 for BaP (n=6), respectively.

***Extraction and analysis of PAHs from soil and the soil slurry***

Residual PAHs from the soil and soil slurry was extracted using a modified ultrasound-assisted solvent extraction method described by (Subashchandrabose *et al.* 2017). Briefly, an equal volume of acetone-hexane (1:1, v/v) was added to dry soil or soil slurry. After vigorous vortexing for 2 min, the contents were sonicated for 15 min (3×, 5 min cycle) in a 360 W ultrasonic water bath (Ultrasonics Australia) with a mean operating frequency of 40 Hz. Then, after centrifugation at 3000 ×*g* for 10 min, the organic fraction was collected. The procedure was repeated thrice; the pooled extract was dried over anhydrous Na_2_SO_4_ and evaporated under a gentle stream of nitrogen. Finally, the residue was reconstituted in 1.0 mL hexane, sonicated briefly, passed through a syringe-driven 0.45 µm pore size PTFE filter, and stored at –20 °C until analysis.

One microlitre sample was injected in splitless mode with an injector temperature of 275 °C. Helium (He) at a constant flow rate of 1.2 mL min^–1­^ was used as the carrier gas. The oven temperature was kept at 40 °C for a min, then raised to 120 °C at 25 °C min^–1^. Later the temperature was increased to 200 °C at 10 °C min^–1^ and further heated up to 300 °C at 5 °C min^–1^ and maintained for 10 min. The total run time was 43.2 min. The mass spectrophotometer source temperature was set at 230 °C. The instrument monitored selective ions while operating in EI mode at 70 eV for all the analytes. He at 2.25 mL min^–1­^ and N_2_ at 1.5 mL min^–1­^ were used as the quench gas and collision gas, respectively, in the collision cell. PAHs in the soil extracts were determined from a standard calibration curve with R^2^ ≥0.99. The calibration solutions were prepared from Sigma-Aldrich’s TraceCERT^®^ quality reference 16 USEPA PAHs calibration mix (Supelco, CRM47940). The reproducibility of the calibration was validated by injecting a standard after every 10 samples and validated if the instrument response was found in 80-120% range. A blank sample was injected after every ten samples to check for carryover; the amount was below detection limits. Triplicate samples from each treatment were analyzed. The extraction steps were carried out in solvent-washed amber glass vials fitted with a screw cap and PTFE-lined septa. The extraction efficiency was calculated based on the recovery of surrogate naphthalene-d_8_, phenanthrene-d_10,_ and chrysene-d_12_ from representative samples (*n* = 6). The surrogates were added to the soil slurry samples prior to extraction and the recoveries ranged from 82 to 105%.

**References**

Caporaso JGK, J. *et al.* (2010) QIIME allows analysis of high-throughput community sequencing data. Nat Methods 7:335-336

Edgar RC (2010) Search and clustering orders of magnitude faster than BLAST. Bioinformatics 26:2460-2461

Quast C, Pruesse E, Yilmaz P, Gerken J, Schweer T, Yarza P, Peplies J, Glockner FO (2013) The SILVA ribosomal RNA gene database project: improved data processing and web-based tools. Nucleic Acids Res 41:D590-596

Subashchandrabose SR, Logeshwaran P, Venkateswarlu K, Naidu R, Megharaj M (2017) Pyrene degradation by *Chlorella* sp. MM3 in liquid medium and soil slurry: Possible role of dihydrolipoamide acetyltransferase in pyrene biodegradation. Algal Res 23:223-232
